# Familial dysalbuminemic hyperthyroxinemia: 2 Indian cases with a novel *ALB* variant (p.Asn415Ile) identified in one

**DOI:** 10.1210/jcemcr/luag026

**Published:** 2026-03-05

**Authors:** Satish Kumar Samal, Shreya Kottapalli, Anusha Nadig, Anusha Handral, Srinivasa Phanidhar Munigoti, Vijaya Sarathi

**Affiliations:** Department of Endocrinology, Vydehi Institute of Medical Sciences and Research Centre, Bengaluru, Karnataka 560066, India; Department of Endocrinology, Vydehi Institute of Medical Sciences and Research Centre, Bengaluru, Karnataka 560066, India; Department of Endocrinology, Vydehi Institute of Medical Sciences and Research Centre, Bengaluru, Karnataka 560066, India; Department of Endocrinology, Manipal Hospital, Bengaluru, Karnataka 560066, India; Department of Endocrinology, Fortis Hospital, Bengaluru, Karnataka 560076, India; Department of Endocrinology, Vydehi Institute of Medical Sciences and Research Centre, Bengaluru, Karnataka 560066, India

**Keywords:** familial dysalbuminemic hyperthyroxinemia, *ALB* p.Asn415Ile, Abbott ARCHITECT

## Abstract

Familial dysalbuminemic hyperthyroxinemia (FDH) is a rare condition caused by pathogenic *ALB* variants, typically involving Arg242. We describe 2 Indian families with FDH. Case 1 showed elevated total (TT4) and free thyroxine (fT4) with normal thyroid-stimulating hormone (TSH). Her 2 children displayed a similar pattern. All 3 had ∼2-6-fold TT4 and 2-4-fold fT4 elevations on Roche COBAS and Beckman ACCESS assays, while Ortho VITROS reported low-normal fT4 and Abbott ARCHITECT showed minimal or no hormone elevation. Genetic analysis identified a novel heterozygous *ALB* variant, p.Asn415Ile. Case 2 was evaluated after markedly high TT4 was detected during routine screening. He demonstrated 11-13-fold TT4 elevation on most assays, except Ortho VITROS, and only a modest increase on Abbott ARCHITECT. Genetic testing confirmed the p.Arg242Ser *ALB* variant. To conclude, we report a novel *ALB* variant and show that Abbott ARCHITECT exhibits the least assay interference among the contemporary immunoassay platforms in FDH.

## Introduction

Familial dysalbuminemic hyperthyroxinemia (FDH) is a rare autosomal dominant disorder caused by pathogenic variants in the *ALB* gene that increase albumin's affinity for thyroxine. Familial dysalbuminemic hyperthyroxinemia most frequently arises due to variants at Arg242 in the *ALB* gene, with p.Arg242His being the most common, followed by p.Arg242Ser and p.Arg242Pro; the p.Arg246Ile variant has been reported only rarely [1, 2]. The prevalence of FDH is ∼0.01% in Caucasians but significantly higher (1.0-1.8%) in Hispanics [3]. Although FDH has been described across multiple populations, no cases have previously been reported from India.

Familial dysalbuminemic hyperthyroxinemia is generally benign, although an association with increased miscarriage risk has been documented [4]. Most affected individuals are detected incidentally during thyroid testing performed for nonspecific indications or routine health evaluations. Because FDH presents with elevated total thyroxine (TT4) and free thyroxine (fT4) in the presence of normal thyroid-stimulating hormone (TSH), it is often mistaken for other causes of euthyroid hyperthyroxinemia, such as excess thyroxine-binding globulin (TBG), or more rarely, a TSH-secreting pituitary adenoma or thyroid hormone resistance [1]. Such misinterpretation frequently leads to unnecessary investigations, and in rare cases, individuals may be incorrectly treated for hyperthyroidism [5, 6].

The magnitude of TT4 and fT4 elevation varies depending on the underlying *ALB* variant and is further influenced by the immunoassay platform used [7]. Although equilibrium dialysis and liquid chromatography provide accurate fT4 measurements in FDH, availability remains limited [1, 8, 9]. Hence, identifying immunoassays with minimal interference is essential. Here, we report 2 Indian cases of FDH, with a novel *ALB* variant in one, and describe the interassay variability in TT4 and fT4 elevations.

## Case presentation

### Case 1

A 61-year-old South Indian woman (FI-I), born of a nonconsanguineous marriage, presented with a 5-year history of temporal headaches. Initial testing showed mildly elevated TSH (5.5 µU/mL; Système International [SI]: 5.5 mU/L) (reference range, 0.35-4.94 µU/mL [SI: 0.35-4.94 mU/L]), for which she was started on levothyroxine 25 μg daily. On endocrine referral, repeat evaluation on Beckman ACCESS revealed TSH 0.83 µU/mL (SI: 0.83 mU/L) (reference range, 0.34-5.6 µU/mL [SI: 0.34-5.6 mU/L]), normal antithyroperoxidase antibodies, and markedly elevated TT4 (29.3 µg/dL; SI: 377.7 nmol/L) (reference range, 5.5-14.3 µg/dL [SI: 70.8-184.04 nmol/L]) and fT4 (5.23 ng/dL; SI: 67.3 pmol/L) (reference range, 0.61-1.12 ng/dL [SI: 7.85-14.41 pmol/L]). Levothyroxine was discontinued, and she remained clinically euthyroid.

### Case 2

A 39-year-old East Indian man (FII-I), born of a nonconsanguineous marriage, was referred after routine testing showed TT4 > 24.77 µg/dL (SI: >318.9 nmol/L) (reference range, 5.1-14.1 µg/dL [SI: 65.6-181.5 nmol/L]) with TSH 3.1 µU/mL (SI: 3.1 mU/L) (reference range, 0.27-4.2 µU/mL [SI: 0.27-4.2 mU/L]) on Roche COBAS e411. He reported mild fatigue but had no features of thyrotoxicosis.

## Diagnostic assessment

### Case 1

Her albumin (3.9 g/dL; SI: 39 g/L) (SI: 39 g/L) (reference range, 3.5-5.0 g/dL [SI: 35-50 g/L]) and TBG (24.1 μg/mL; SI: 371.14 nmol/L) (reference range, 14.0-31.0 μg/mL [SI: 215.6-477.4 nmol/L]) were normal. Pituitary magnetic resonance imaging (MRI) did not suggest a TSH-secreting adenoma. Whole exome sequencing, to evaluate for the genetic causes of euthyroid hyperthyroxinemia, identified a novel heterozygous *ALB* variant, p.Asn415Ile (c.1244A>T) in exon 10 (variation ID: 3344578, accession: VCV003344578.1). The variant was absent from 1000 Genomes, gnomAD (v2 and v3.1), TOPMed, and internal databases. PolyPhen-2 (HumDiv), Sorting Intolerant From Tolerant, and Likelihood Ratio Test predicted it to be probably damaging/damaging, and the codon was evolutionarily conserved. No other rare variants in *ALB*, *TTR*, or *THRB* were detected.

Family testing showed abnormal thyroid profiles in both sons (FI-II and FI-III) (Table 1), but not in her brother or mother. Sanger sequencing confirmed the variant in the proband and her sons, supporting likely pathogenicity. Across assay platforms, TT4 was elevated on Ortho VITROS (4.1-6.4-fold), Roche COBAS (2.1-2.8-fold), and Beckman ACCESS (2.1-2.4-fold). fT4 was elevated on Roche COBAS (3.1-3.9-fold) and Beckman ACCESS (2.0-2.2-fold) but low/normal on Ortho VITROS. Abbott ARCHITECT showed only marginal elevations.

**Table 1:** Thyroid function tests of Case 1 (mother), her 2 affected sons, and Case 2 in various contemporary immunoassay platforms

		Ortho VITROS® 5600	Beckman ACCESS UniCel Dxl 800	Roche COBAS e411	Abbott ARCHITECT i2000SR
TT3	FI-I	1.29 ng/mL (SI: 1.99 nmol/L)	1.87 ng/mL (SI: 2.88 nmol/L)	2.6 ng/mL (SI: 4.0 nmol/L)	1.29 ng/mL (SI: 1.99 nmol/L)
	FI-II	1.55 ng/mL (SI: 2.39 nmol/L)	2.03 ng/mL (SI: 3.13 nmol/L)	1.72 ng/mL (SI: 2.65 nmol/L)	1.24 ng/mL (SI: 1.9 nmol/L)
	FI-III	1.54 ng/mL (SI: 2.37 nmol/L)	1.78 ng/mL (SI: 2.74 nmol/L)	1.77 ng/mL (SI: 2.73 nmol/L)	1.49 ng/mL (SI: 2.3 nmol/L)
	FII-I	1.96 ng/mL (SI: 3.01 nmol/L)	2.08 ng/mL (SI: 3.2 nmol/L)	2.02 ng/mL (SI: 3.11 nmol/L)	1.9 ng/mL (SI: 2.93 nmol/L)
	RR	0.4-1.8 ng/mL (SI: 0.61-2.77 nmol/L)	0.7-2.0 ng/mL (SI: 1.07-3.08 nmol/L)	0.8-2.0 ng/mL (SI: 1.23-3.08 nmol/L)	0.7-2.0 ng/mL (SI: 1.07-3.08 nmol/L)
TT4	FI-I	43.3 μg/dL*^a^* (SI: 553.4 nmol/L)	>30.2 μg/dL (SI: >388.6 nmol/L)	29.3 μg/dL*^a^* (SI: 377.1 nmol/L)	11.1 μg/dL (SI: 142.9 nmol/L)
	FI-II	67.5 μg/dL*^a^* (SI: 868.7 nmol/L)	34.1 μg/dL*^a^* (SI: 438.6 nmol/L)	36.7 μg/dL*^a^* (SI: 472.33 nmol/L)	11.8 μg/dL (SI: 151.86 nmol/L)
	FI-III	49.5 μg/dL*^a^* (SI: 637.06 nmol/L)	29.8 μg/dL (SI: 383.5 nmol/L)	39.4 μg/dL*^a^* (SI: 507.1 nmol/L)	11.6 μg/dL (SI: 149.3 nmol/L)
	FII-I	139 μg/dL*^a^* (SI: 1788.93 nmol/L)	186.5 μg/dL*^a^* (SI: 2400.25 nmol/L)	151.6 μg/dL*^a^* (SI: 1951.1 nmol/L)	21.65 μg/dL (SI: 278.63 nmol/L)
	RR	4.6-10.5 μg/dL (SI: 59.2-135.13 nmol/L)	5.5-14.3 μg/dL (SI: 70.78-181.46 nmol/L)	5.1-14.1 μg/dL (SI: 65.63-181.46 nmol/L)	4.5-11.0 μg/dL (SI: 57.91-141.57 nmol/L)
fT3	FI-I	2.99 pg/mL (SI: 4.57 pmol/L)	5.55 pg/mL (SI: 8.54 pmol/L)	10.45 pg/mL (SI: 16.1 pmol/L)	3.72 pg/mL (SI: 5.72 pmol/L)
	FI-II	4.87 pg/mL (SI: 7.5 pmol/L)	5.9 pg/mL (SI: 9.1 pmol/L)	10.5 pg/mL (SI: 16.17 pmol/L)	3.95 pg/mL (SI: 6.1 pmol/L)
	FI-III	4.55 pg/mL (SI: 7.01 pmol/L)	5.2 pg/mL (SI: 8.1 pmol/L)	10.2 pg/mL (SI: 15.7 pmol/L)	4.03 pg/mL (SI: 6.21 pmol/L)
	FII-I	5.13 pg/mL (SI: 7.9 pmol/L)	5.72 pg/mL (SI: 8.8 pmol/L)	9.43 pg/mL (SI: 14.5 pmol/L)	5.3 pg/mL (SI: 8.16 pmol/L)
	RR	2.77-5.27 pg/mL (SI: 4.23-8.01 pmol/L)	2.5-3.9 pg/mL (SI: 3.85-6.0 pmol/L)	2.0-4.4 pg/mL (SI: 3.08-6.77 pmol/L)	2.3-4.2 pg/mL (SI: 3.54-6.46 pmol/L)
fT4	FI-I	0.89 ng/dL (SI: 11.45 pmol/L)	2.34 ng/dL (SI: 30.12 pmol/L)	5.23 ng/dL (SI: 67.31 pmol/L)	1.32 ng/dL (SI: 16.98 pmol/L)
	FI-II	0.95 ng/dL (SI: 12.2 pmol/L)	2.4 ng/dL (SI: 30.88 pmol/L)	6.7 ng/dL (SI: 86.23 pmol/L)	1.57 ng/dL (SI: 20.2 pmol/L)
	FI-III	0.65 ng/dL (SI: 8.36 pmol/L)	2.21 ng/dL (SI: 28.44 pmol/L)	5.21 ng/dL (SI: 67.05 pmol/L)	1.18 ng/dL (SI: 15.18 pmol/L)
	FII-I	1.09 ng/dL (SI: 14.02 pmol/L)	6.02 ng/dL (SI: 77.48 pmol/L)	5.41 ng/dL (SI: 69.62 pmol/L)	1.8 ng/dL (SI: 23.16 pmol/L)
	RR	0.78-2.19 ng/dL (SI: 10.3-28.19 pmol/L)	0.61-1.12 ng/dL (SI: 7.85-14.41 pmol/L)	0.93-1.70 ng/dL (SI: 11.96-21.88 pmol/L)	0.70-1.48 ng/dL (SI: 9.0-19.04 pmol/L)
TSH	FI-I	2.98 μU/mL (SI: 2.98 mU/L)	2.83 μU/mL (SI: 2.83 mU/L)	3.74 μU/mL (SI: 3.74 mU/L)	2.85 μU/mL (SI: 2.85 mU/L)
	FI-II	0.9 μU/mL (SI: 0.9 mU/L)	0.98 μU/mL (SI: 0.98 mU/L)	1.12 μU/mL (SI: 1.12 mU/L)	1.33 μU/mL (SI: 1.33 mU/L)
	FI-III	1.04 μU/mL (SI: 1.04 mU/L)	1.2 μU/mL (SI: 1.2 mU/L)	1.24 μU/mL (SI: 1.24 mU/L)	0.79 μU/mL (SI: 0.79 mU/L)
	FII-I	0.51 μU/mL (SI: 0.51 mU/L)	1.82 μU/mL (SI: 1.82 mU/L)	1.85 μU/mL (SI: 1.85 mU/L)	1.53 μU/mL (SI: 1.53 mU/L)
	RR	0.5-8.9 μU/mL (SI: 0.5-8.9 mU/L)	0.34-5.6 μU/mL (SI: 0.34-5.6 mU/L)	0.27-4.2 μU/mL (SI: 0.27-4.2 mU/L)	0.35-4.94 μU/mL (SI: 0.35-4.94 mU/L)

Abbreviations: FI-I, Case 1; FI-II and FI-III, sons of Case 1; FII-I, Case 2; fT3, free triiodothyronine; fT4, free thyroxine; RR, reference range; TSH, thyroid-stimulating hormone; TT3, total triiodothyronine; TT4, total thyroxine.

^*a*^Tested in 1:10 dilution.

### Case 2

Repeat testing on Beckman ACCESS showed free triiodothyronine (fT3) 5.07 pg/mL (SI: 6.53 pmol/L), (reference range, 2.5-3.9 pg/mL [SI: 3.22-5.01 pmol/L]), fT4 5.62 ng/dL, and TSH 2.98 µU/mL (SI: 3.1 mU/L). Albumin (4.1 g/dL; SI: 41 g/L) and TBG (19.3 μg/mL; SI: 297.2 nmol/L) were normal. He reported that his mother had similar results, though she was unavailable for evaluation. Whole exome sequencing identified the known pathogenic p.Arg242Ser (c.724C>A) *ALB* variant. TT4 was markedly elevated (10.8-13-fold) on Roche COBAS, Beckman ACCESS, and Ortho VITROS. fT4 was elevated (3.2-5.4-fold) on Roche COBAS and Beckman ACCESS but normal on Ortho VITROS. Abbott ARCHITECT showed substantially lower elevations (TT4 ∼2-fold; fT4 ∼1.2-fold).

## Treatment

Both probands and the affected children were counseled about the benign nature of FDH and the assay-related false elevations of TT4 and fT4.

## Outcome and follow-up

One year later, Case 1 received an urgent laboratory alert for a “critically high” TT4 value. Knowing her diagnosis, she reassured the laboratory and continued her routine care without unnecessary interventions.

## Discussion

We describe 2 Indian cases of FDH, including a novel likely pathogenic *ALB* variant, p.Asn415Ile, in Case 1. Of the 4 thyroxine-binding sites on albumin, subdomains IIA and IIIA have the highest affinity. Variants at Arg242 in subdomain IIA are well-established causes of FDH [10]. The novel variant in Case 1 represents the first reported alteration in subdomain IIIA. Structural studies show that in subdomain IIIA, the outer ring of T4 forms hydrogen bonds with Tyr425 and Ser513, while the inner ring interacts via van der Waals forces with Gln414, Asn415, Leu418, Ala430, and Arg434 [10]. Substitution at Asn415 likely disrupts these interactions, increasing thyroxine affinity; functional studies are needed to confirm pathogenicity. Case 2 harbored p.Arg242Ser, previously reported in several families, including one from Bangladesh [4, 6, 11].

The extent of TT4 and fT4 elevation varies across *ALB* variants. Individuals with p.Arg242His (up to 1.7-fold) and p.Arg246Ile (1.99-fold) show milder TT4 elevation, whereas p.Arg242Ser (up to 13.3-fold) and p.Arg242Pro (up to 16.6-fold) exhibit higher levels [6, 7, 11-14]. A similar pattern occurs for fT4, with modest increases in p.Arg242His (up to 2.46-fold) and p.Arg246Ile (2.32-fold) and much higher levels in p.Arg242Ser (up to 8.7-fold) and p.Arg242Pro (up to 11.7-fold) [6, 7, 11-13]. The 3.2-5.4-fold fT4 and 10.8-13-fold TT4 rise in Case 2 (p.Arg242Ser) align with published data. Case 1's family showed 2-3.9-fold fT4 and 2-6.4-fold TT4 elevations—greater than p.Arg242His but lower than p.Arg242Ser.

In patients with euthyroid hyperthyroxinemia, a normal fT4 favors the diagnosis of TBG excess and excludes the diagnosis of TSH-secreting adenoma and thyroid hormone resistance (THRB inactivation). In FDH, fT4 is elevated in most of the contemporary immunoassays, which often confuses the condition with the latter 2 conditions. Although a family history prompted early genetic evaluation in Case 2, it followed MRI pituitary in Case 1. Notably, all 4 FDH individuals in our study had elevated TT4 and fT4 on Roche and Beckman platforms but low-normal or low fT4, despite high TT4, on the Ortho VITROS. Hence, in individuals with euthyroid hyperthyroxinemia, an elevated fT4 in other immunoassays but low-normal or low fT4 in Ortho VITROS should indicate a diagnosis of FDH. Elevation of total triiodothyronine (TT3) and fT3 was also often observed in individuals with FDH but disproportionately lower compared to those with fT4, which reflects a lower affinity of T3 to albumin [15].

Platform-specific variability in fT4 measurement is well recognized [6, 7]. In FDH patients with p.Arg242His, fT4 elevations are lower on PerkinElmer DELFIA and Abbott ARCHITECT than on Beckman ACCESS, Siemens CENTAUR, Roche ELECSYS, and Fujirebio LUMIPULSE [7]. Although 2-step methods are considered less susceptible to interference, markedly elevated fT4 levels on the Beckman ACCESS platform—a 2-step assay—challenge this assumption. Given that fT4 represents only ∼0.03% of TT4, even minor alterations in T4-protein binding equilibrium can substantially affect estimated fT4 concentrations [1]. Maintaining optimal pH and temperature is essential to preserve equilibrium between bound and free T4 in vitro. Platforms using chloride-containing buffers (eg, Beckman ACCESS) report significantly higher fT4, whereas assays with chloride-free media (eg, Ortho VITROS) show lower levels. Adding chloride increases fT4 in Ortho VITROS, and increasing chloride concentration elevates fT4 even when measured by equilibrium dialysis. However, elevated fT4 is also observed in some chloride-free assays (Roche COBAS), while certain chloride-containing assays (PerkinElmer DELFIA, pH 7.8) demonstrate less pronounced effects [7], indicating additional assay-related influences, particularly pH [15]. Roche COBAS uses a phosphate buffer—known to minimally affect T4–albumin binding at pH 7.4—but the assay operates at pH 7.0, which may significantly reduce binding of T4 to variant albumin in FDH without affecting wild-type albumin [16]. Binding of T4 to albumin and mutant albumin is also inhibited, though to a lesser degree, by barbital [15, 17, 18], potentially explaining high fT4 levels with Siemens CENTAUR [7]. The use of a (chloride-free) Tris buffer may contribute to the milder elevations seen in Abbott ARCHITECT. Although chloride and pH largely account for interassay differences, additional factors likely contribute and warrant further investigation.

Equilibrium dialysis and liquid chromatography-tandem mass spectrometry (LC-MS/MS) remain the gold standards for fT4 measurement in FDH. Among immunoassays, Abbott ARCHITECT is the least prone to assay interference with the highest concordance with LC-MS/MS in FDH patients with p.Arg242His [9, 11, 19 , 20]. In FDH individuals with p.Arg242Ser also, minimal assay interference has been reported with Abbott ARCHITECT [6, 11]. Similarly, Case 2 with p.Arg242Ser also had minimal assay interference in Abbott ARCHITECT. We also report no or minimal interference for fT4 measurement in Abbott ARCHITECT in the novel FDH variant with p.Asn41Ile. However, Abbott ARCHITECT is prone to substantial assay interference in FDH individuals with p.Arg242Pro, the variant with largest fT4 elevation [12, 14].

Notably, TT4 elevation was also less pronounced on Abbott ARCHITECT. Although TT4 was increased 2-fold in Case 2 (p.Arg242Ser), the rise was only marginal in individuals carrying the p.Asn415Ile variant, who typically show fT4 and TT4 concentrations that fall between those observed in p.Arg242His and p.Arg242Ser. A similar pattern has previously been described in an individual with p.Arg242His [21]. The relatively modest TT4 elevation with Abbott ARCHITECT may reflect a weaker capacity of this assay to displace T4 bound to variant albumin, in contrast to platforms such as Roche COBAS and Beckman ACCESS, both of which utilize potent displacement agents like 8-anilino-1-naphthalene sulfonic acid [17]. Conversely, the higher TT4 values recorded on Ortho VITROS may be related to its use of a barbital buffer; however, unlike its TT4 assay, the fT4 assay on Ortho VITROS does not contain barbital.

## Learning points

- We report a novel *ALB* variant, p.Asn415Ile, associated with the FDH phenotype, thereby expanding the known genetic spectrum of FDH.
- Individuals carrying the p.Asn415Ile variant show intermediate elevations of TT4 and fT4 compared with those harboring p.Arg242His and p.Arg242Ser.
- In patients with euthyroid hyperthyroxinemia, the combination of elevated fT4 in most immunoassays but low-normal or low fT4 on the Ortho VITROS platform strongly suggests FDH and helps distinguish it from thyroid hormone resistance and TSH-secreting pituitary adenoma.
- The degree of TT4 and fT4 elevation in FDH is influenced by both the underlying genotype and the immunoassay platform, with the Abbott ARCHITECT platform showing the least assay interference.

## Data Availability

Data sharing is not applicable to this article as no datasets were generated or analyzed during the current study.
